# Multifocal acceptance score to evaluate vision: MAS-2EV

**DOI:** 10.1038/s41598-021-81059-0

**Published:** 2021-01-14

**Authors:** Xoana Barcala, Maria Vinas, Mercedes Romero, Enrique Gambra, Juan Luis Mendez-Gonzalez, Susana Marcos, Carlos Dorronsoro

**Affiliations:** 1 2Eyes Vision, Madrid, Spain; 2 Institute of Optics, Spanish National Research Council, IO-CSIC, Madrid, Spain

**Keywords:** Quality of life, Health care

## Abstract

We present a new metric (Multifocal Acceptance Score, MAS-2EV) to evaluate vision with presbyopic corrections. The MAS-2EV is based on a set of images representing natural visual scenes at day and night conditions projected in far and near displays, and a near stereo target. Subjects view and score the images through different binocular corrections (monofocal corrections at far; bifocal corrections; monovision and modified monovision) administered with soft contact lenses (in cyclopleged young subjects) or with a binocular simultaneous vision simulator (in presbyopic and cyclopleged young subjects). MAS-2EV scores are visually represented in the form of polygons, and quantified using different metrics: overall visual quality, visual degradation at far, visual benefit at near, near stereo benefit, visual imbalance near-far, overall visual imbalance and a combined overall performance metric. We have found that the MAS-2EV has sufficient repeatability and sensitivity to allow differentiation across corrections with only two repetitions, and the duration of the psychophysical task (3 min for subject/condition/correction) makes it useable in the clinic. We found that in most subjects binocular bifocal corrections produce the lowest visual imbalance, and the highest near stereo benefit. 46.67% of the subjects ranked binocular bifocal corrections first, and 46.67% of the subjects ranked monovision first. MAS-2EV, particularly in combination with visual simulators, can be applied to select prospective presbyopic corrections in patients prior to contact lens fitting or intraocular lens implantation.

## Introduction

Presbyopia, the loss of the ability to focus near objects, affects 100% of the population over 45 years old^1^. There is an increasing number of presbyopic corrections, beyond standard near vision spectacles of progressive addition, which include monovision and multifocal solutions, in the form of contact lenses (CLs), intraocular lenses (IOLs) or refractive surgery (RS).

In monovision, one eye is corrected for far distance and the other for near distance to provide functional vision at all distances. However, for a given distance at least one eye is out of focus. Monovision relies on a selective suppression of the defocused images coming from one eye while the brain uses the images from the eye that is in focus. Despite some disadvantages caused by the interocular blur difference, as the loss of binocular vision and stereopsis^2^, monovision correction is still the most popular spectacle-free presbyopic correction^3^.

Alternatively, simultaneous vision^4^ creates a superposition of image components with different amounts of defocus but similar content, position and magnification. Typically, simultaneous vision IOLs (multifocal IOLs; M-IOLs) are based on diffractive optics, refractive optics or a hybrid approach, while multifocal CLs (M-CLs)^5^ tend to be based on rotationally-symmetric zonal refractive designs, with a part of the pupil devoted for far, and another for near (either in the center or periphery). There is an increasing number of M-IOLs and M-CLs in the market. In general, functionality at near and far (sometimes also intermediate distances) is achieved at the expense of some reduction in visual quality (blur and contrast reduction) at all distances^6^.

Typically, the quality of vision provided by multifocal lenses, monovision and combined strategies (i.e. modified monovision where the dominant eye is corrected with a monofocal lens at far and the non-dominant eye is corrected with a multifocal lens^3^) is tested using visual acuity (VA) at various distances or using defocus curves^7–10^. It is well recognized that high contrast VA is a limited descriptor of the quality of vision. Besides, the complexity and unfamiliarity of multifocal vision^11^ require wider and most sophisticated evaluation methods.

Quality of vision is, in fact, multifactorial, and largely depends on visual conditions that affect luminance, pupil diameter, and on the spatial content and contrast of the visual world^12^.

To date, the quality of vision is assessed clinically by means of questionnaires that are given to the patient who self-report visual comfort and task performance in different situations (i.e. reading a restaurant menu, driving, sewing, etc.…)^13^. Examples of these questionnaires include NEI RQL-42^14^, a questionnaire with 42 questions that measure the patient’s satisfaction at distance vision, clarity of vision, and severity and frequency of glare symptoms, along with the need for spectacles. Other questionnaires also include an assessment of the satisfaction with day and night vision (as the Functional Assessment of Visual Tasks (VISTAS) questionnaire^15,16^) or with night driving (as VF-14, the Visual Function Index). Some questionnaires (i.e. The Catquest-9SF questionnaire) address the perceived benefits of cataract surgery^17^. While the previous questionnaires were not specifically designed to evaluate the quality of vision with multifocal lenses, the CLUE questionnaire^18^ developed by Johnson and Johnson targets specifically the quality of vision (as well as other comfort aspects) with M-CLs.

Drawbacks associated to vision questionnaires have been addressed before. For example, systematic psychometric evaluations showed that the NEI-RQL-42 questionnaire has deficiencies in most of its tested aspects^19^. All questionnaires, even those optimally designed, rely on the patient’s memory, as they rate their vision for situations that they encountered over different days. Furthermore, the evaluations provided by the questionnaires may differ across patients, as not every patient is exposed to the same visual environment.

In this paper, we present for the first time a perceived visual quality test (Multifocal Acceptance Score to Evaluate Vision, MAS-2EV) that combines the systematicity and accessibility of those tests conducted using visual displays in the clinic (such a VA), with the multi-component description of the visual world that is captured in the questionnaires. In MAS-2EV, natural images representing scenes that can be encountered in daily life (daytime and nighttime, near and far distances, and stereovision) are scored by the patient.

The metric can be applied to patients already implanted with a M-IOL, or fitted with CLs of various designs, similarly to the indicated VA tests or questionnaires. However, the real value of the metric relies on the possibility to perform these tests pre-operatively or before fitting real CLs on eye. Pre-operative or pre-fitting simulations are now possible with the use of visual simulators.

Adaptive optics visual simulators typically based on deformable mirrors or spatial light modulators have been used to replicate various multifocal lens designs^7,20–23^. Alternatively, SimVis Gekko (SimVis, 2EyesVision), a see-through wearable binocular visual simulator^8,24,25^ with a wide field of view (> 20°) is targeted to prospective M-IOL and M-CLs patients. SimVis simulates multifocal corrections using tunable lenses working under temporal multiplexing. Temporal multiplexing is based on fast periodic optical power variations at speeds greater than the defocus flicker fusion of the human visual system, generating on the patient’s retina multifocal images that are apparently static and programmable^7,26,27^.

The new Multifocal Acceptance Score to Evaluate Vision (MAS-2EV) presented here is ideally administered in combination with binocular visual simulators such as SimVis. The patient experiences through different presbyopic corrections (simulating prospective contact or intraocular lenses) realistic daytime and nighttime natural scenes at near and at far, representative of different situations that the patient may encounter in his/her daily life (with different illuminations, distances, contrasts, glare sources and spatial content). We evaluated the repeatability and sensitivity of the metric in differentiating across different corrections, using both the SimVis and real CLs on eye.

## Materials and methods

### Multifocal acceptance score to evaluate vision (MAS-2EV)

MAS-2EV is a custom-developed metric defined as a multi-component vector, comprising five perceptual scores (PS; 0–10) of multi-stimuli images of day and night scenes, at far (4 m) and near (40 cm) distance, and of a stereovision target at near. The perceived image quality of the global visual scene at far and near distances through a given correction was judged by the subject using a perceptual scoring technique^6^ ranging from very blurred (score 0) to very sharp (score 10).

**Figure 1:**
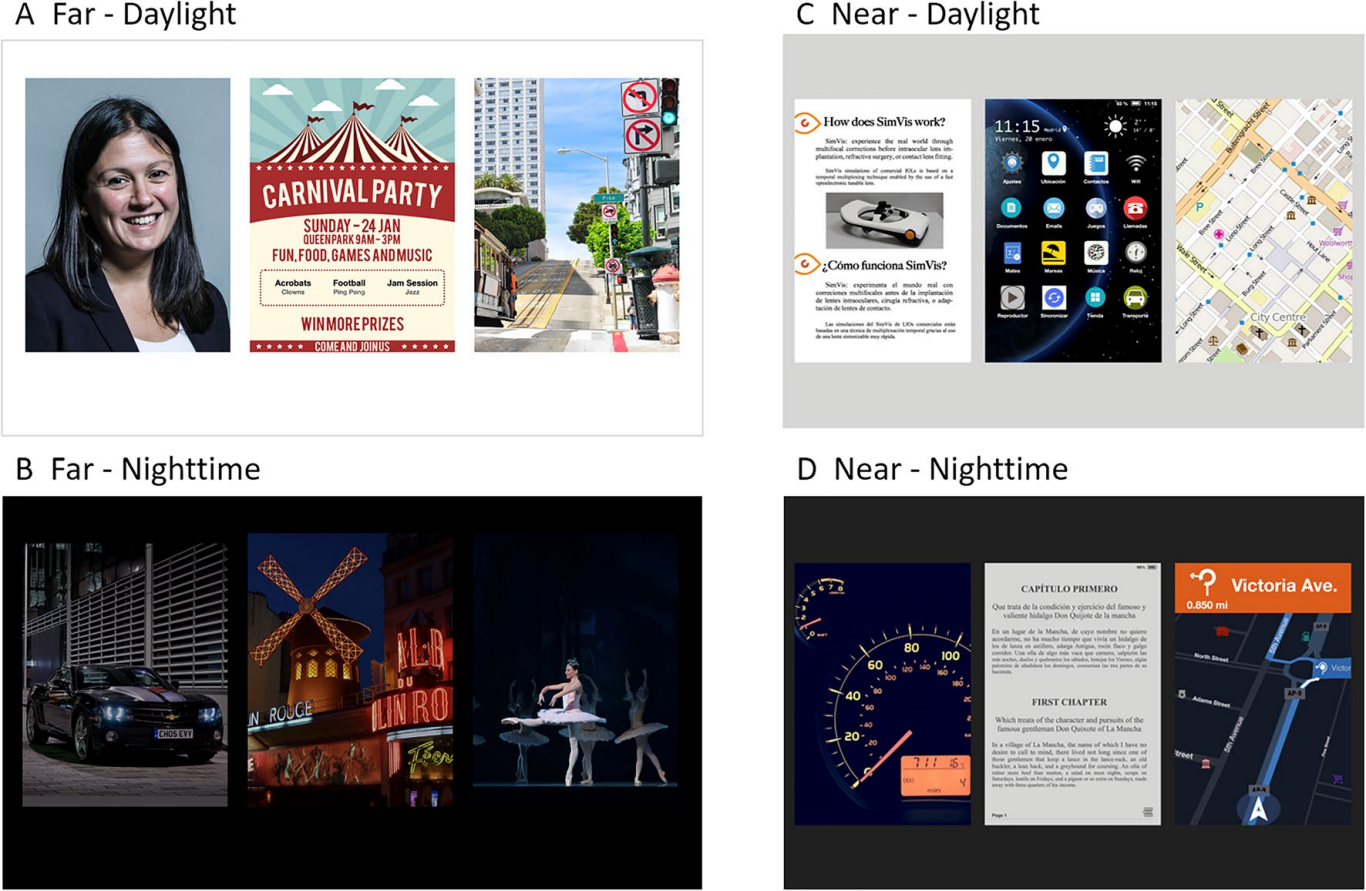
Set of images of MAS-2EV (CC BY open access license, further details in Acknowledgments and Attributions section).

The selected images, shown in Fig. 1, represent four different daily-life visual activity areas: far-day, far-night, near-day, and near-night. The images are available to distribute and either come from public repositories of images (details of the sources, licenses and attributions can be found on the acknowledgments) or have been specifically generated for this study.

The far-day image (Fig. 1A) set consists of a female portrait; a poster with different letter fonts and sizes; and an urban street view. The far-night set (Fig. 1B) represents urban scenes at night: a view of a stopped car with the lights on and a readable license plate, with a pair of bright superimposed white LEDs (18 cd/m^2^) simulating real headlights introducing glare, a street scene with neon signs of different sizes; and an indoor ballet show. Near-day (Fig. 1C) activities are represented by a flyer with different text sizes; a smartphone screen with app icons and text; and a digital city map. The near-night (Fig. 1D) set consisted of a car’s glowing dashboard at night; an electronic book screenshot with low contrast and different letter sizes; and a GPS navigation map in night mode. The power spectra of the face and street scene images followed roughly the 1/spatial frequency of natural images with a spatial frequency range between < 0.25 and > 40 cpd. The objects shown were designed to be seen at a specific distance and to represent habitual visual scenes for a patient, the size of the text in the images ranged from 0 to 1 logMAR VA. The contrast and color of each image was carefully selected for match with the light condition and distance.

Additionally, near stereo-acuity targets are presented, consisting of a Random-dot anaglyph with seven in-depth Snellen-E letters with different orientations and different crossed disparities (400–50 arcsec). For the stereo test, the score corresponds to the number of letters whose orientation the subject can detect at 40 cm, ranging from 0, for stereo disparities equal or above 400 arcsec, to 10, for a disparity of 50 arcsec. The anaglyphs are presented in an iPad Pro 12.9″ with Retina Display (by Apple Inc) and observed with cyan/red glasses.

The MAS-2EV stimuli were designed to be presented on a standard monitor screen of 42″ for far distance, and on standard iPad screen of 9.8″, being possible a resize to fit them on higher screens without modifying the size of the stimuli. In this study, they were presented on a 48.5″ display (3840 × 2160 pixels, 49UH850V, LG) at 4 m for far distance and on an iPad Pro (2732 × 2048 pixels) at 40 cm for near distance. With the displays switched off, the room illumination was above 400 lx for day-time conditions, and bellow 2.5 lx for night-time conditions. The average luminance of the MAS-2EV stimuli presented on the displays was measured with a colorimeter ColorCal (MKII, Cambridge Research Systems). The average luminances were 120 ± 51.21 cd/m^2^ (for far-day), 131.67 ± 17.28 cd/m^2^ (for near-day), 5.40 ± 5.79 cd/m^2^ (far-night) and 1.86 ± 0.30 cd/m^2^ (near-night). The brightness control of the displays was not changed between day and night conditions for far distance. The brightness control of the iPad was changed from the maximum (day condition) to the minimum (night condition) for near distance.

### Subjects

Measurements were performed in ten healthy subjects: five young subjects (age: 29 ± 6 years; spherical error (sph): -2.23 ± 2.07 D) and five presbyopic subjects (age: 61 ± 4; 1.20 ± 0.45D). Astigmatism was lower than 0.75 D in all subjects. Anisometropia was lower than 1.50 D in all subjects. All presbyopic subjects wore reading glasses, but they were spectacle-free for far distance. Presbyopia was pharmacologically simulated in young subjects by instillation of one drop of 1% tropicamide 15 min before the measurements, and then every hour. The accommodation amplitude (A.A.) in presbyopic subjects was measured using a Power Refractor 2 (PlusOptix, Germany), and was 1.35 ± 0.33 D, on average. The subject profiles are shown in Table 1 with individual data of age, gender, sphero-cylindrical refraction, eye dominance and accommodation amplitude.

**Table 1:** Individual data of gender (_M male; _F Female); age (years); Sphero-cylindrical refraction (Rx, sph and cyl in D, x astigmatic angle in degrees) in right (OD) and left (OS) eyes; eye dominance (OD right eye; OS left eye) and amplitude of accommodation (A.A, in D for OD/OS, only in presbyopes).

Subject	Age	Rx OD (Sph, cyl)	Rx OS (Sph, cyl)	Dominance	A.A. OD/OS
S1_ F	26	0.00, − 0.50 × 30	− 0.25, − 0.50 × 150	OD	Cyclopleged
S2_F	26	− 3.75, − 0.50 × 30	− 5.00, − 0.50 × 65	OD	Cyclopleged
S3_F	29	− 2.00, − 0.25 × 95	− 2.50, − 0.25 × 40	OD	Cyclopleged
S4_M	24	− 4.00, − 0.50 × 35	− 4.75, − 0.50 × 165	OS	Cyclopleged
S5_F	38	0.00, − 0.75 × 180	0.00, − 0.75 × 170	OD	Cyclopleged
S6_M	57	+ 0.25	+ 0.75, − 0.50 × 70	OS	1.56/1.30
S7_F	68	+ 1.25, − 0.25 × 80	+ 1.50, − 0.25 × 80	OS	0.93/1.07
S8_F	59	+ 1.00, − 0.75 × 100	+ 1.00, − 0.75 × 80	OS	1.61/0.93
S9_F	60	+ 1.50, − 0.50 × 55	+ 1.50, − 0.50 × 105	OS	1.29/1.92
S10_F	61	+ 1.75, − 0.50 × 80	+ 1.50, + 0.25 × 55	OS	1.27/1.67

### Experiments

All young subjects were measured both with CLs and SimVis. Presbyopic subjects only performed measurements with SimVis. Subjects were divided in three different groups: (1) young subjects measured with SimVis; (2) young subjects measured with CLs; (3) presbyopic subjects measured with SimVis. The experimental protocol was the same for all groups.

The experiments conformed to the tenets of the Declaration of Helsinki, with protocols approved by the Consejo Superior de Investigaciones Cientificas Ethics Committee. The subjects signed an informed consent after receiving an explanation of the nature and implications of the study.

### Tested binocular presbyopic corrections

Four binocular presbyopic corrections were tested, both with CLs and simulated using SimVis in young subjects, and SimVis in presbyopic subjects: (1) Monofocal far in both eyes (FF); (2) Bifocal lenses in both eyes (BB); (3) Monovision (FN; dominant eye with monofocal far and non-dominant eye with monofocal near); and (4) Modified Monovision (FB; dominant eye with monofocal far and non-dominant eye with a bifocal lens). The near add in bifocal corrections was + 2.50 D, and the interocular refraction difference was + 2.50 D in the monovision correction.

### Contact lenses

In one experiment, patients wore monofocal and M-CLs (Biofinity, CooperVision, USA)^28^. The lenses were silicone hydrogel (Comfilcon A) with standard geometry parameters (Base Curve: 8.6, Lens diameter: 14.0) and monthly disposable. Distance power ranged from − 0.25 to − 5.00 D in both monofocal and bifocal lenses. M-CLs had a central near add of + 2.50 D, with an aspheric front surface and a mono-curve back surface.

CLs were fitted following the manufacturer’s guide and checked for lens damage under the slit-lamp before proceeding with lens settling. Evaluation of the lens centration, primary gaze movement, upgaze movement, and tightness was carried out following routinary standard practice^29^. CLs had a settling time of at least 4 min before proceeding.

### SimVis Gekko

A SimVis Gekko v0.5 visual simulator^8,24,25^ was used in this study. This wearable device has the capability to simulate, in either eye, programmable multifocal corrections. In this study, we used generic multifocal profiles with an energy distribution of 50% for far distance (0.00 D) and 50% for near distance (+ 2.50 D) programmed in the SimVis. The bifocal lens design was produced using a temporal multiplexing technique ^24^. Trial lenses inserted in a dedicated slot in the system were used to correct the far distance refraction in the multifocal designs (from − 1.00 D to + 4.50 D, in 0.25-diopter steps) and to replicate a monofocal correction.

The device was calibrated using a high-speed focimeter^26^ to measure both the optical power and the dynamic effects of the optotunable lens. The lens was driven according to that calibration, including, for the simulation of the bifocal lenses, the compensation of the dynamic effects measured^30^. The calibration of the device was checked weekly to guarantee the stability of the simulation throughout the study. It should be noted that the lens designs used in this study correspond to generic monofocal and bifocal (pure simultaneous vision) designs, and do not aim at reproducing the specific contact lens designs of “Contact lenses”.

### Experimental protocol

The measurements were conducted by two experienced optometrists. Conventional non-cyclopleged subjective refraction was obtained using Optonet Vision Unit (Optonet Ltd, United Kingdom).

The patient’s sphero-cylindrical refraction was corrected with the spherical equivalent by the CLs power (in the measurements with CLs) or by trial lenses placed in the dedicated slot in the SimVis device. Natural binocular vision was tested using a 4-dot Worth test to discard fusion dysfunction, which was an exclusion criterion. Eye dominance was determined by the + 1.50 D blur test.

A preparatory trial using SimVis allowed the subject to set the range for their perceptual scoring by viewing the MAS-2EV far-day images through a simulated far-distance corrected monofocal lens (10 PS) and an additional + 2.50 D monofocal lens (0 PS).

**Figure 2:**
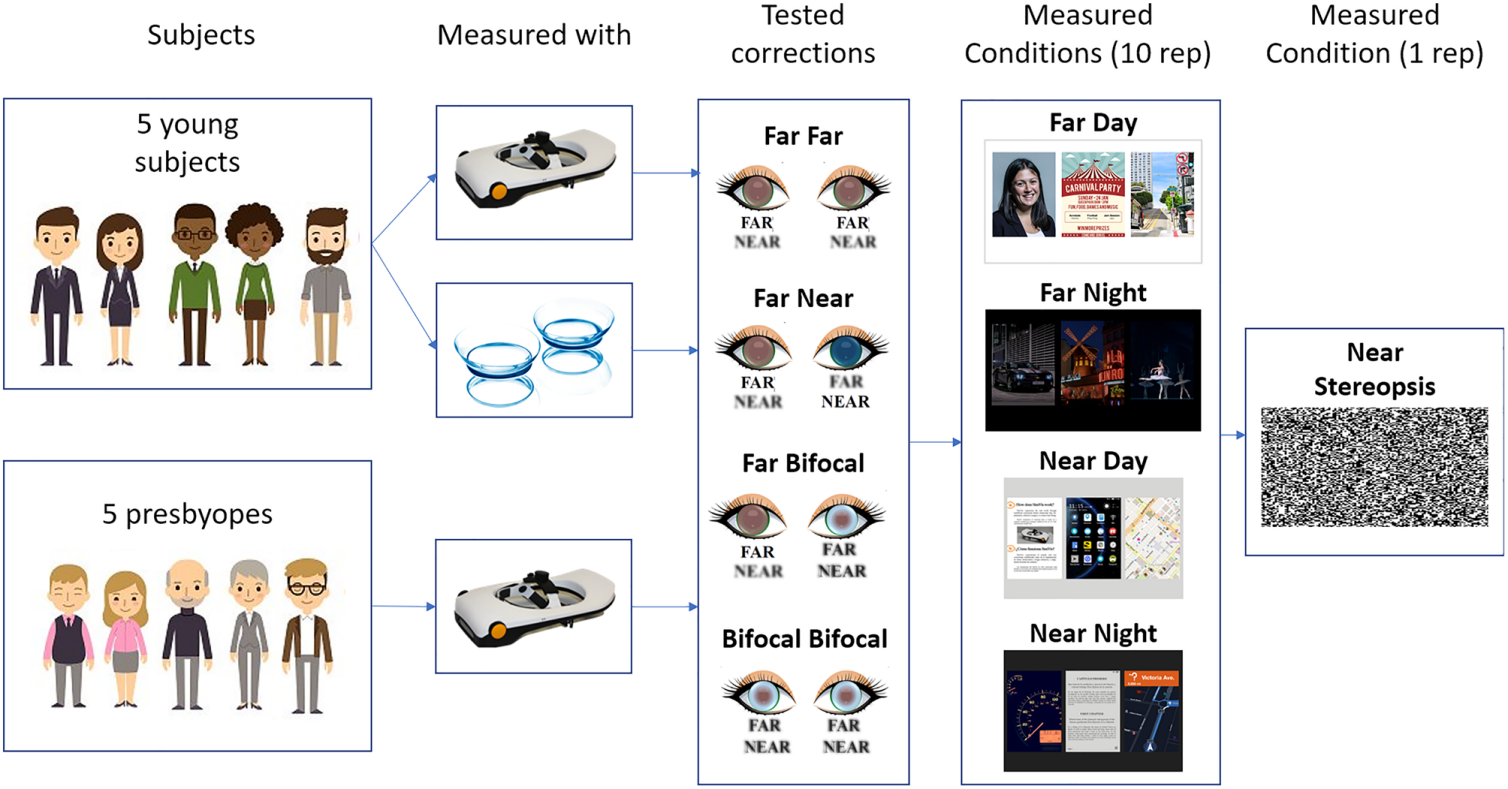
Illustration summarizing the methods, showing the subjects measured divided into three groups depending on the simulator/contact lens, the corrections tried by each group, and the measured conditions. Further explanations in "Subjects", "Experiments", "Tested binocular presbyopic corrections" and "Experimental protocol" (CC BY open access license, further details in Acknowledgments and Attributions section).

Figure 2 illustrates and summarized the methodology followed in the study. The set of four presbyopic corrections described in “Tested binocular presbyopic corrections” was tested first with the SimVis in the young and presbyopic subjects, and then with CLs only in young subjects (in different sessions). Within each method, corrections were tested following a preset random series.

For a given correction, the stereopsis at near was first tested (yielding a 0–10 score), according to the description in “Multifocal acceptance score to evaluate vision (MAS-2EV)”. Then MAS-2EV scorings were first obtained for far-day and near-day. Patients were then allowed to settle into the night-time setting for 4 min, after which MAS-2EV scorings were obtained for far-night and near-night.

A total of 1640 trials were conducted in young subjects (SimVis and CLs), and 820 trials in presbyopic subjects (SimVis), corresponding to 4 corrections, 2 light conditions, 2 distances, each repeated 10 times, and 4 stereo responses. A full MAS-2EV task takes 2 min to be completed per correction. Measurements were performed in two different experimental sessions, lasting 5 h (SimVis) or 10 h (CLs).

### Data analysis and visualization

MAS-2EV is graphically represented as a polygon, with the Perceptual Scores (PS) in each vertex: the measured stereopsis (upper vertex) and the four PS measured for far-day, far-night, near-day, and near-night (an example is shown in Fig. 2). The geometrical center of the polygon corresponds to score 0 and the maximum separation of the vertex to score 10.

A metric of the overall visual quality with a specific correction is given by the unweighted average (MAS-2EV Modulus) of the five vertices. Alternatively, the overall visual quality can be calculated as the normalized area enclosed by the MAS-2EV polygon (MAS-2EV Area), defined as: (*area* − *area_min*)/(*area_max* − *area_min*) where *area* is the area enclosed by the MAS-2EV polygon for a given subject and correction, *area_max* is the maximum area obtained across subjects and corrections, and the *area_min* the minimum area obtained across subjects and corrections.

The visual imbalance across distances (near and far) for a given correction was obtained by subtracting the PS at near from the PS at far. Additionally, the overall visual imbalance across conditions (near/far, day/night, stereo) for a given correction was calculated as the standard deviation across all PS (normalized to the maximum) and represented the asymmetry of the MAS-2EV polygons.

Metrics of visual compromise were also obtained from the MAS-2EV scores, in relation to the PS for the bilateral monofocal corrections (FF): (1) Visual degradation at far, defined as: (*PS Far for each presbyopic correction* – *PS Far FF*)/*PS Far FF*; (2) Visual benefit at near, defined as: (*PS Near for each presbyopic correction* – *PS Near FF*)/*PS Far FF*; (3) Near stereo benefit is defined as: (*Stereo with FF* – *Stereo for each presbyopic correction*)/*Best Near Stereo* for that subject.

The intra-subject repeatability was calculated as the mean of the standard deviation across repetitions for each subject. Inter-subject differences were calculated with the standard deviation of the MAS-2EV Modulus.

Statistical analysis was performed in IBM SPSS Statistics v26 to (1) to analyze the statistical relevance of each variable (subject, correction, distance, and illumination) using a Mixed Model Analysis: Main effect and all two-way; (2) analyze the reliability and the consistency in the scoring criteria used by the subjects with an Alpha Cronbach; (3) analyze the significance of the difference between two variables across the same group using a Related-Samples Friedman's 2-way ANOVA by Ranks with pairwise comparison-adjusted by the Bonferroni correction; (4) analyze the significance of a variable between groups using an Independent-Samples Kruskal–Wallis Test with pairwise comparison adjusted by Bonferroni correction; (5) analyze the repeatability of the metric using a Repeated Measures ANOVA analysis between factors.

## Results

**Figure 3:**
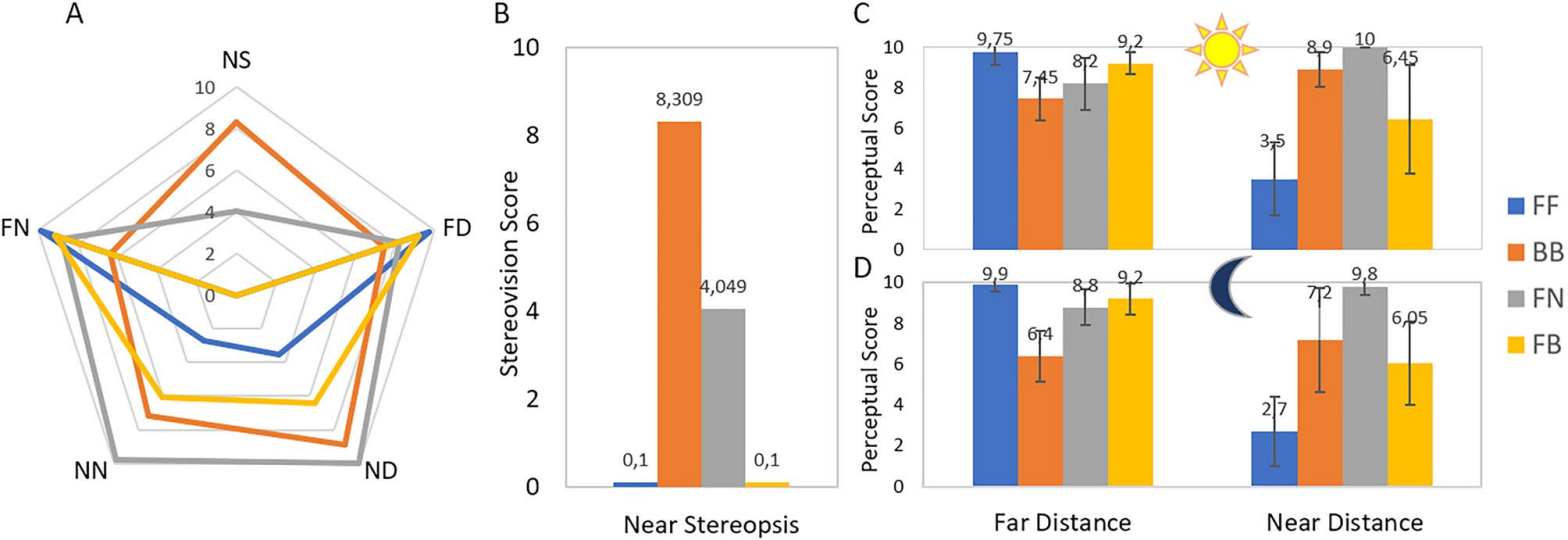
MAS-2EV polygons, near stereovision and perceptual scores for subject S1_SV, with presbyopic corrections simulated with SimVis. (**A**) MAS-2EV polygon. The upper vertices represent stereopsis score (NS), and the four lower vertices perceptual scores for far-day (FD), near-day (ND), near-night (NN) and far-night (FN) counter-clockwise. (**B**) Near stereovision scores. (C and D) Perceptual scores (PS) for far distance (left columns) and near distance (right columns), for day stimuli (upper graph) and night stimuli (lower graph) for the different corrections. In all graphs Blue stands for both eyes corrected for far (FF); Orange both eyes corrected with bifocal lenses (+ 2.50 D add, BB); Gray stands for monovision (monofocal for far distance in the dominant eye and + 2.50 D monofocal in the non-dominant eye, FN); Yellow stands for modified monovision (monofocal for far distance in the dominant eye and bifocal lens with + 2.50 D near add in the non-dominant eye, FB). Each data point is the average across 10 repeated measurements, and error bars represent standard deviations.

**Figure 4:**
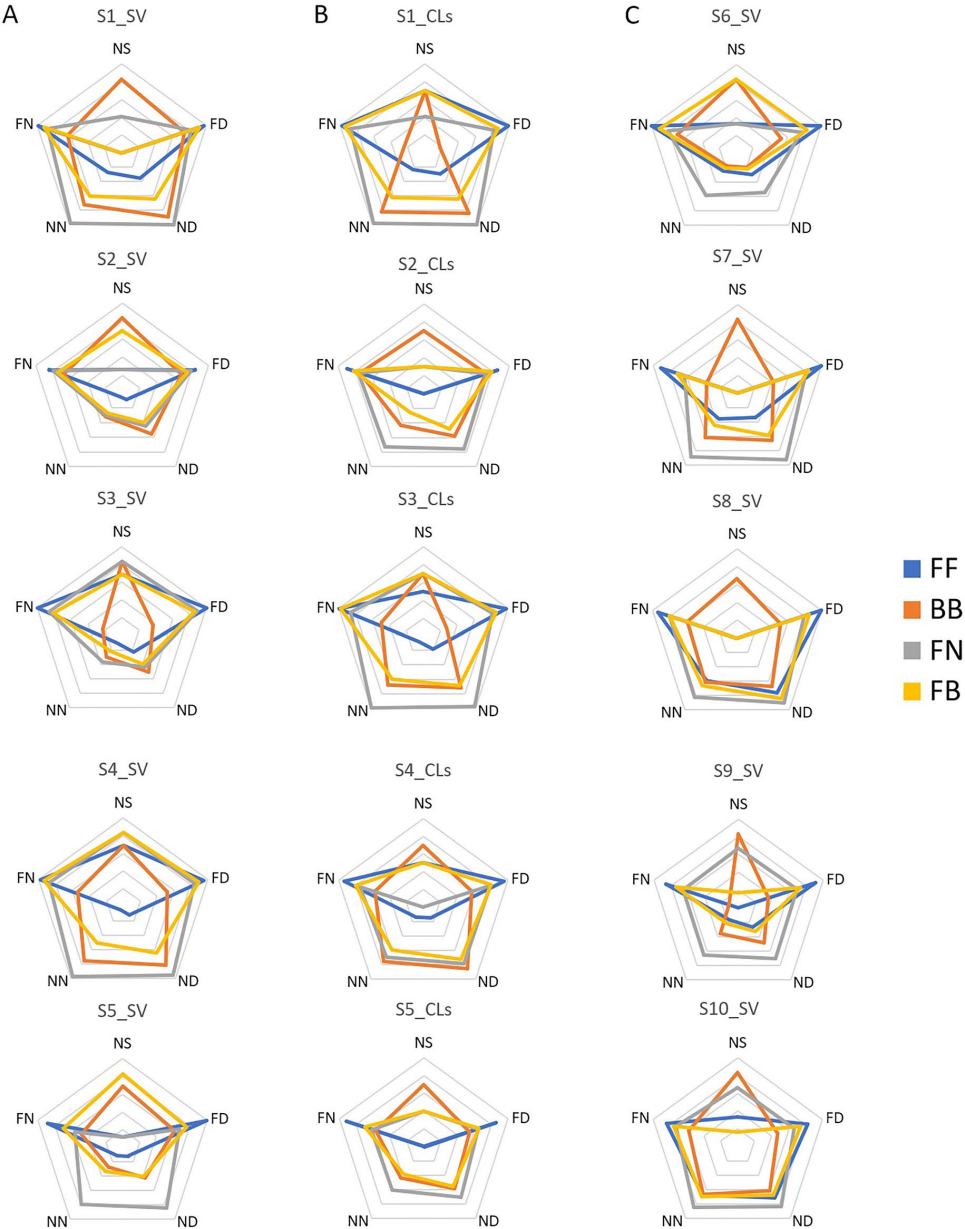
MAS-2EV polygons. (**A**) For young subjects measured with SimVis. (**B**) For young subjects measured with CLs. (**C**) For presbyopic subjects measured with SimVis. Blue line corresponds to bilateral monofocal correction for far (FF), orange line to bilateral bifocal (BB), gray line to monovision (FN), and yellow line to modified monovision (FB).

**Figure 5:**
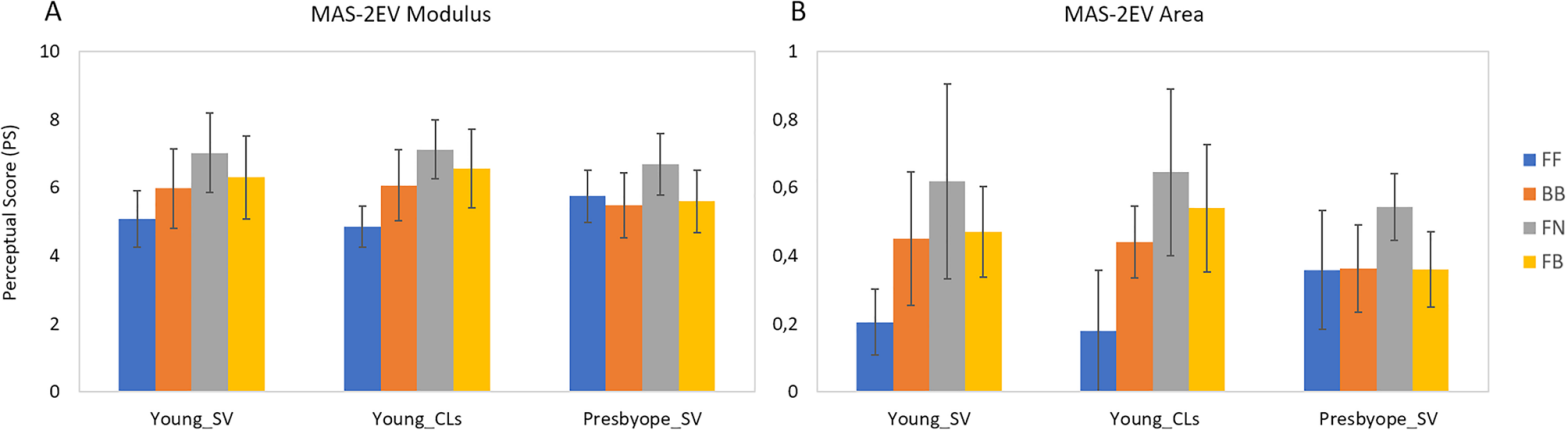
(**A**) MAS-2EV Modulus (average of the five polygon vertices: NS and the four PS averaged across subjects for each group), for all corrections. (**B**) MAS-2EV Area (normalized areas averaged across subjects for each group), for all corrections.

**Figure 6:**
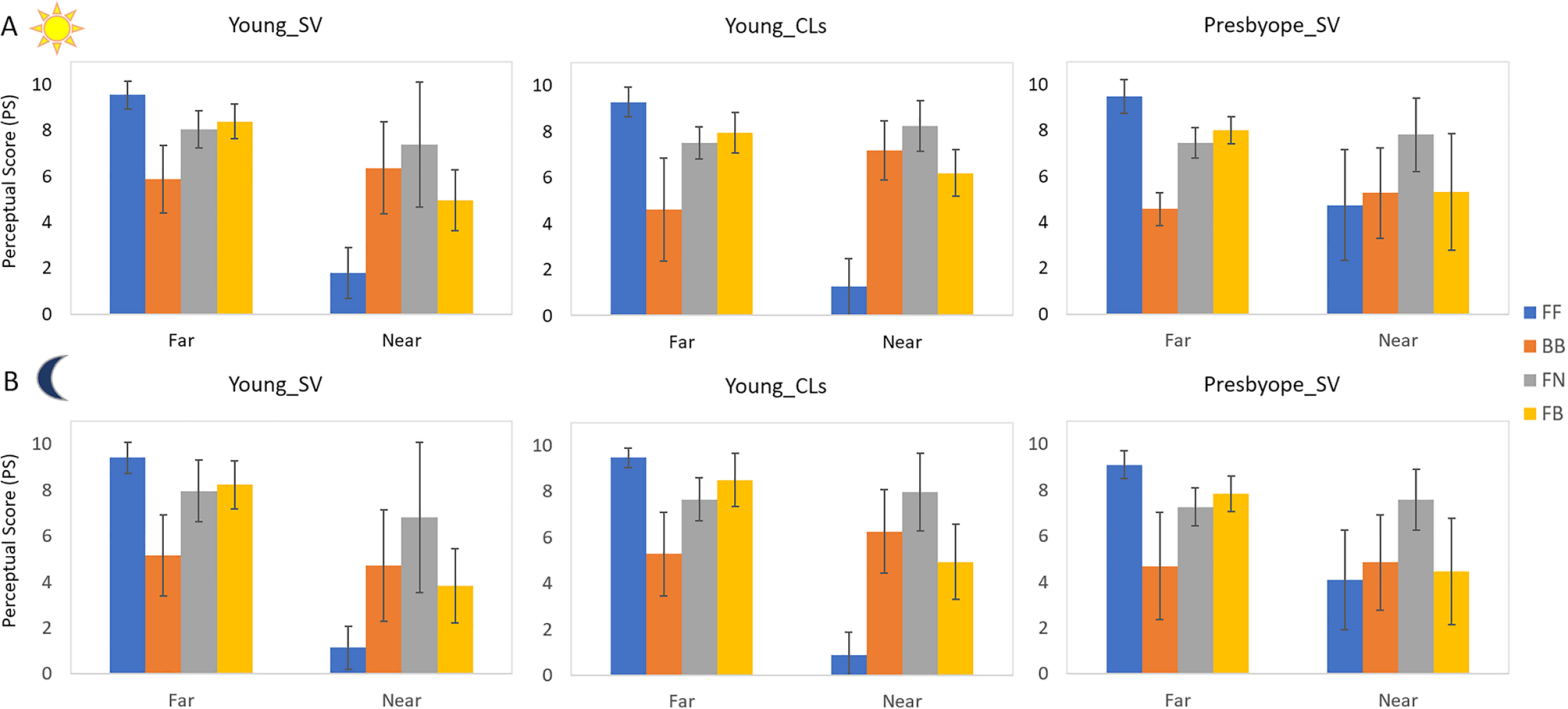
Mean PS across subjects for day (**A**, upper row), night (**B**, lower row) conditions, at far and near distances.

**Figure 7:**
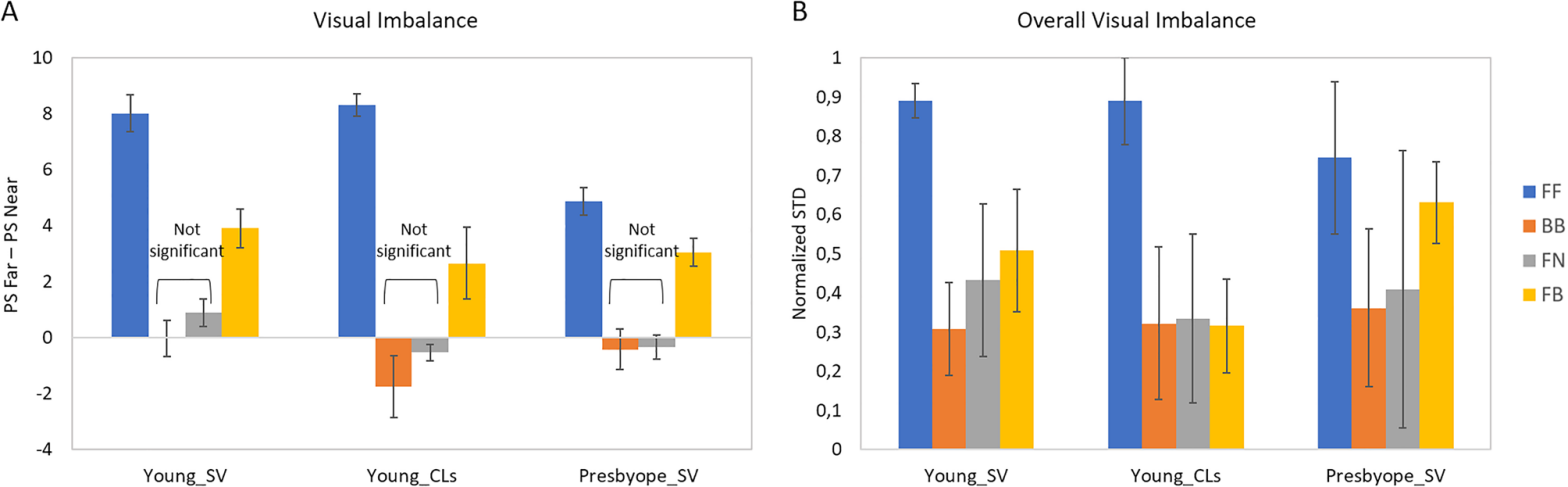
(**A**) Visual imbalance between distances (difference PS Far-PS Near) for each correction and group (averaged across light conditions and subjects). (**B**) Overall visual imbalance (normalized standard deviation in PS, averaged across subjects) for each for each correction and group.

**Figure 8:**
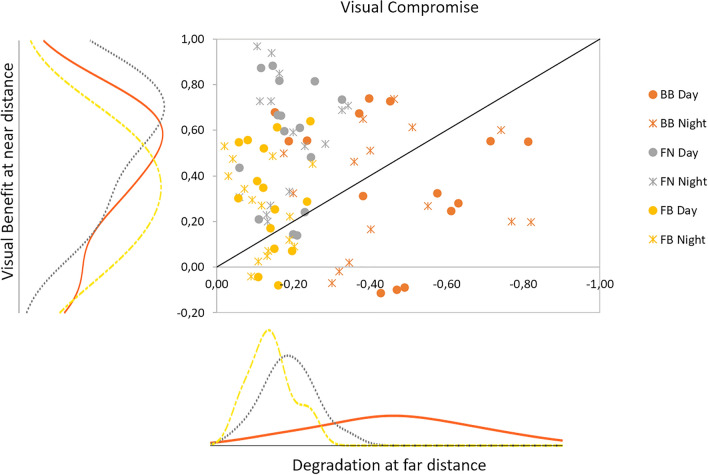
Visual compromise: visual degradation at far vs visual benefit at near, with respect to a bilateral monofocal correction (FF), for all presbyopic corrections (bilateral bifocal BB, monovision FN, modified monovision FB). Symbols represent each subject measured at day (spots) and night (asterisks) conditions. The histograms show the distribution of the data for each axis.

**Figure 9:**
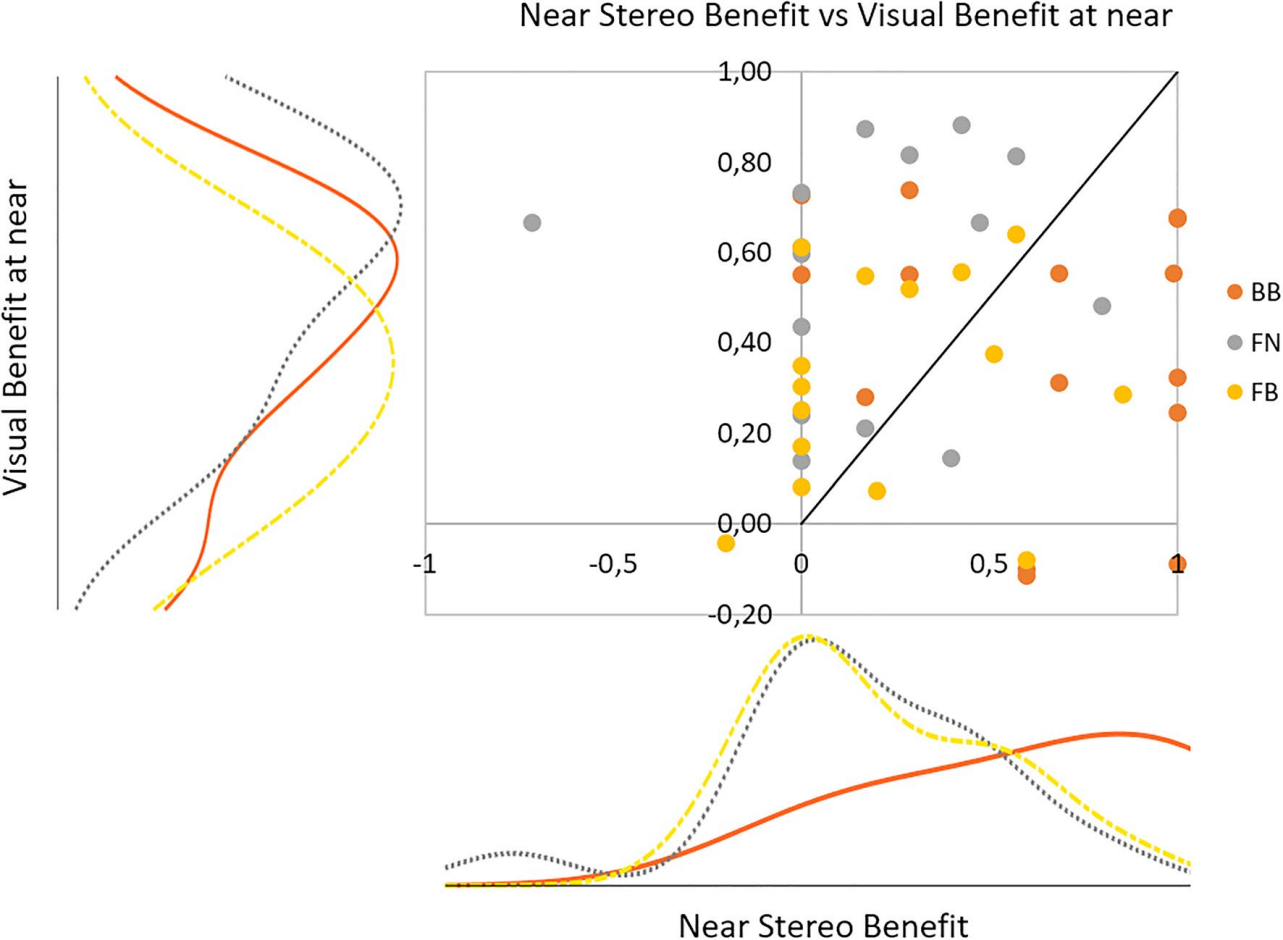
Near stereo benefit as a function of Visual Benefit at near for each presbyopic correction. The histograms show the distribution for each axis.

 “Analysis of the variables and reliability of the metric” presents the statistical analysis and relevance of each variable, as well as the reliability of the metric. “MAS-2EV polygons, near stereopsis and perceptual scores” presents (1) an example of how to construct the MAS-2EV polygon that represents the ten perceptual score repetitions for each subject (Fig. 3); (2) all MAS-2EV polygons for all subjects (Fig. 4) showing differences across corrections and groups; (3) MAS-2EV Modulus and Areas averaged across subjects (Fig. 5) and; (4) a comparison between day and night conditions (Fig. 6). “Visual imbalance and compromise of different presbyopic corrections” presents: (1) visual imbalance for each correction (Fig. 7); (2) visual compromise across distances (Fig. 8); (3) near stereo benefit (Fig. 9); (4) overall visual imbalance and; (5) overall visual quality metrics (Tables 2 and 3). “Inter- and intrasubject variability” presents the MAS-2EV inter- and intra- subject variability. Finally, “Metric repeatability” shows the repeatability of the proposed metric.

**Table 2:** Individual data of visual degradation at far distance, visual benefit at near distance, near stereo benefit, overall visual imbalance (negative), overall visual quality, overall performance parameter and ranking.

Subject	Correction	Degradation @Far	Benefit @Near	Benefit stereo	− Overall visual imbalance	Overall visual quality	Overall performance parameter	Ranking
S1_SV	FF	0.00	0.00	0.00	− 0.91	0.24	− 0.67	4
	**BB**	− 0.30	0.51	0.99	− 0.16	0.73	**1.77**	**①**
	**FN**	− 0.14	0.70	0.48	− 0.48	0.82	**1.38**	2
	FB	− 0.06	0.32	0.00	− 0.76	0.44	− 0.06	3
S2_SV	FF	0.00	0.00	0.00	− 0.83	0.10	− 0.73	4
	**BB**	− 0.19	0.44	0.68	− 0.37	0.45	**1.01**	**①**
	**FN**	− 0.06	0.37	0.00	− 0.52	0.31	**0.1**	3
	**FB**	− 0.11	0.32	0.51	− 0.44	0.38	**0.66**	2
S3_SV	FF	0.00	0.00	0.00	− 0.87	0.33	− 0.54	4
	BB	− 0.70	0.24	0.17	− 0.47	0.23	− 0.53	3
	**FN**	− 0.13	0.24	0.17	− 0.50	0.53	**0.31**	**①**
	FB	− 0.17	0.15	0	− 0.55	0.40	− 0.17	2
S4_SV	FF	0	0	0	− 0.92	0.24	− 0.68	4
	**BB**	− 0.46	0.73	0	− 0.23	0.54	**0.58**	3
	**FN**	− 0.11	0.92	0.17	− 0.09	1.00	**1.89**	**①**
	**FB**	− 0.05	0.51	0.17	− 0.35	0.70	**0.98**	2
S5_SV	FF	0	0	0	− 0.94	0.11	− 0.83	4
	**BB**	− 0.39	0.24	0.68	− 0.30	0.30	**0.53**	2
	**FN**	− 0.33	0.71	0	− 0.58	0.42	**0.22**	3
	**FB**	− 0.21	0.25	0.85	− 0.44	0.43	**0.88**	**①**
S6_SV	FF	0	0	0	− 0.79	0.32	− 0.47	4
	BB	− 0.39	− 0.09	0.60	− 0.59	0.24	− 0.23	3
	**FN**	− 0.21	0.29	0	− 0.37	0.44	**0.15**	**①**
	**FB**	− 0.13	− 0.06	0.60	− 0.72	0.36	**0.05**	2
S7_SV	FF	0	0	0	− 0.86	0.24	− 0.62	4
	**BB**	− 0.56	0.30	1.00	− 0.36	0.40	**0.78**	**①**
	**FN**	− 0.23	0.57	0	− 0.78	0.48	**0.04**	2
	FB	− 0.18	0.17	0	− 0.65	0.31	− 0.35	3
S8_SV	FF	0	0	0	− 0.82	0.51	− 0.31	4
	**BB**	− 0.42	− 0.04	1.00	− 0.10	0.46	**0.9**	**①**
	FN	− 0.17	0.19	0	− 0.76	0.53	− 0.21	2
	FB	− 0.14	0.08	0	− 0.73	0.48	− 0.31	3
S9_SV	FF	0	0	0	− 0.86	0.16	− 0.7	4
	**BB**	− 0.72	0.22	1.00	− 0.52	0.22	**0.2**	2
	**FN**	− 0.24	0.51	0.80	− 0.00	0.57	**1.64**	**①**
	FB	− 0.16	0.06	0.20	− 0.56	0.20	− 0.1	3
S10_SV	**FF**	0	0	0	− 0.40	0.57	**0.17**	3
	**BB**	− 0.37	− 0.07	0.60	− 0.24	0.50	**0.42**	2
	**FN**	− **0.17**	**0.17**	**0.40**	− **0.14**	**0.70**	**0.96**	**①**
	FB	− 0.11	− 0.01	− 0.20	− 0.49	0.44	− 0.37	4

Corrections in bold indicate acceptable overall performance. Circled correction **①** indicates the highest ranking. Data correspond to measurements performed with SimVis.

**Table 3:** Individual data of visual degradation at far distance, visual benefit at near distance, near stereo benefit, overall visual imbalance (negative), overall visual quality, overall performance parameter and ranking.

Subject	Correction	Degradation @Far	Benefit @Near	Benefit stereo	− Overall visual imbalance	Overall visual quality	Overall performance parameter	Ranking
S1_CL	FF	0	0	0	− 0.74	0.42	− 0.32	3
	BB	− 0.78	0.58	0	− 0.64	0.32	− 0.52	4
	**FN**	− 0.18	0.67	0	− 0.17	0.87	**1.19**	**①**
	**FB**	− 0.08	0.37	0	− 0.27	0.70	**0.72**	2
S2_CL	FF	0	0	0	− 0.99	0.00	− 0.99	4
	**BB**	− 0.16	0.59	1.00	− 0.24	0.50	**1.69**	**①**
	**FN**	− 0.15	0.87	0.43	− 0.39	0.53	**1.29**	2
	**FB**	− 0.09	0.43	0.43	− 0.53	0.32	**0.56**	3
S3_CL	FF	0	0	0	− 0.89	0.24	− 0.65	4
	**BB**	− 0.61	0.58	0.29	− 0.36	0.39	**0.29**	3
	**FN**	− 0.15	0.88	0.29	− 0.22	0.95	**1.75**	**①**
	**FB**	− 0.07	0.53	0.29	− 0.28	0.72	**1.19**	2
S4_CL	FF	0	0	0	− 0.83	0.24	− 0.59	4
	**BB**	− 0.39	0.69	0.29	− 0.21	0.59	**0.97**	**①**
	FN	− 0.18	0.63	− 0.71	− 0.69	0.43	− 0.52	3
	**FB**	− 0.15	0.55	0.00	− 0.24	0.59	**0.75**	2
S5_CL	FF	0	0	0	− 1.00	0.00	− 1	4
	**BB**	− 0.39	0.59	1.00	− 0.16	0.40	**1.44**	**①**
	**FN**	− 0.30	0.76	0.57	− 0.21	0.44	**1.26**	2
	**FB**	− 0.25	0.55	0.57	− 0.26	0.36	**0.97**	3

Corrections in bold indicate acceptable overall performance. Circled correction **①** indicates the highest ranking. Data correspond to measurements performed with CLs.

### Analysis of the variables and reliability of the metric

A Mixed Model Analysis (Dependent: PS; Fixed Factors: distance, light condition, correction; Random: Subject; Repeated: distance, light condition, correction, repetition) reveals that all main effects were statistically significant (subject p < 10^–10^; correction p < 10^–10^; distance p < 10^–10^; light condition p < 0.01) in all groups, and the two-way effects involving distance (near/far) were also significant (p < 0.05) in all groups.

The reliability and the consistency in the scoring criteria used by the subjects was high (Cronbach's Alpha > 0.95 for each group, distance, and light condition).

### MAS-2EV polygons, near stereopsis and perceptual scores

Figure 3 shows an example of MAS-2EV polygons (Fig. 3A), near stereovision (NS) scores (Fig. 3B), and individual perceptual scores (PS, Fig. 3C,D) in one subject (S01) for four presbyopic corrections (FF in blue, BB in orange, FN in grey, FB in yellow) simulated by SimVis. Data are averaged across ten repeated measurements. Figure 3C shows the average individual PS for far-day (Fig. 3C, left columns) and near-day (Fig. 3C, right columns) and far-night (Fig. 3D, left columns) and near-night (Fig. 3D, right columns). As expected, the FF correction (Monofocal far in both eyes; blue columns) provided better scores at far distance than at near distance (62.5%). For BB (bifocal lenses in both eyes; orange), the scores were 23% lower than FF, but similar at both distances (6.92 and 8.05, respectively). For FN (Monovision; gray) the scores were high at both distances (65% higher than FF for near). For FB (Modified monovision; yellow) the scores were higher for far distance than for near distance (27.5%), and more balanced across distances than with FF. Although the trends for day and night PS were similar, scores were on average 4.25% higher for day than for night.

Figure 4 shows the MAS-2EV polygons for all measured subjects and conditions: young subjects with SimVis (Fig. 4A, left column), young subjects with CLs (Fig. 4B, middle column) and presbyopic subjects with SimVis (Fig. 4C, right column).

In all subjects, FF correction (blue line) provided the highest PS at far distance for day and night (PS = 9.38 ± 0.73), which was 75.27% on average higher than at near distance (PS = 2.32 ± 2.40). NS (near stereopsis) with FF was low in almost all subjects (NS = 2.76 ± 2.96). Unlike FF, which produced similar polygons across subjects, BB bilateral correction (orange line) produced a larger inter-subject variability in the response (1.71 PS). NS with BB correction (NS = 7.58 ± 0.72 PS) was 63.59% higher than with FF. The FN correction (grey line) provided good visual quality at near distance (PS = 7.64 ± 2.27) for all subjects except S2_SV and S3_SV, and in general, good quality at far distance (PS = 7.66 ± 0.87). However, FN seriously compromised NS (NS = 3.38 ± 2.87 PS, on average) in 12 out of 15 subjects (except in S3_SV, S9_SV, and S10_SV, whose NS was not affected). The FB (yellow line) was nearly as good as FF for far (only 12.3% lower). However, FB was 26.4% higher than FF for near. FB compromised NS (NS = 4.28 ± 3.17 PS) in all subjects (except in S6_SV), although NS with FB was 20.05% higher than FF and 11.87% higher than FN, but 43.54% lower than BB. Despite common trends across subjects, differences in both the shape and the area polygons between subjects suggest different responses to different corrections depending on the subject.

An average metric for vision quality was calculated as the unweighted average (MAS-2EV Modulus, Fig. 5A) of the PS scores and the polygons normalized area (MAS-2EV Area, Fig. 5B). The similarity of the individual polygons for SimVis and CLs in most of the subjects indicates that SimVis captures similar trends as the real CLs, even if SimVis was not programmed to mimic the actual design of the lens.

Both in young and presbyopic subjects, the FN correction produced the best overall visual quality (highest MAS-2EV Modulus and Area). The FF correction produced a significantly lower MAS-2EV Modulus (p < 0.03; paired-sample t-test) than FN and FB in young subjects, but not in presbyopic subjects. The difference between the best and the worst correction was FN-FF = 1.94 ± 1.00 for the young group with SimVis, FN-FF = 2.26 ± 0.74 for the young group with CLs, and FN-BB = 1.21 ± 0.93 for presbyopes. The overall visual quality (MAS-2EV Area) was lowest for FF (0.25 ± 0.16), followed by BB (0.42 ± 0.14), FB (0.46 ± 0.16) and highest (with higher STD) for FN (0.60 ± 0.21).

Figure 6 shows the mean PS across subjects and distances, itemized by group, far/near and day/night conditions. For each group, FF correction was statistically significantly different than BB at far distance both at day and night condition (p = 0.001; Related-Samples Friedman's 2-way ANOVA by Ranks with pairwise comparison-adjusted by the Bonferroni correction). However, FF was statistically significantly different than BB at near distance (both at day and night condition) only for young subjects, both with SimVis (p = 0.02; Related-Samples Friedman's 2-way ANOVA by Ranks with pairwise comparison-adjusted by the Bonferroni correction) and CLs (p = 0.042; same test). Very consistently, PS for FF at near was lower in young (by 71.27%, p < 0.04 -for both illuminations-; Independent-Samples Kruskall-Wallis with pairwise comparison adjusted by Bonferroni correction) than in presbyopic subjects. PS were almost identical for day and night (the average PS difference across distances, corrections and subjects was 0.32).

### Visual imbalance and compromise of different presbyopic corrections

Figure 7A shows the visual imbalance across distances, calculated as far-near PS difference (averaged across subjects and day/night conditions), for each correction. FF and FB corrections provided statistically significant imbalances between far and near distances in all groups (p < 0.005; paired-sample t-test), although those were significantly lower for presbyopes than for young subjects. The lowest far-near imbalances (not statistically different from zero) occurred in all subjects for BB and FN. On average, the largest overall visual imbalance (Fig. 7B) was found for FF (0.84 ± 0.12) followed by FB (0.48 ± 0.13) and FN (0.39 ± 0.25), with the lowest visual imbalance found for BB (0.33 ± 0.17).

Figure 8 shows the visual compromise (degradation at far distance versus visual benefit at near distance) for each correction and each subject. The ideal correction will lie in the (0, 1) coordinate, i.e. low degradation at far and high benefit at near. Points above the -1:1 line are indicative of a positive compromise (more benefit at near than degradation at far).

Out of the 30 evaluations (3 groups × 5 subjects × 2 light conditions) per correction represented in Fig. 8, 28 showed a positive compromise for FN, 20 for FB and 13 for BB. Figure 8 also shows histograms representing the distribution of degradation at far (horizontal axis) and benefit at near (vertical axis) for the three presbyopic corrections. FN produced the highest benefit at near distance across subjects and light conditions (56.38 ± 25.79%; with a peak at of the histogram at 0.69); followed by BB (36.79 ± 27.60%; peak at 0.57), and by FB (28.10 ± 20.89%; peak at 0.33). The degradation at far distance was lowest for FB (− 12.03 ± 5.71%; peak at − 0.14); followed by FN (− 18.26 ± 7.00%; peak at − 0.19); and it was highest for BB (− 45.51 ± 18.38%; peak at − 0.47).

An important aspect not reflected in visual quality at near and far distances is the stereo vision, the fifth parameter in the MAS-2EV metric. Figure 9 shows the near stereo benefit as a function of visual benefit at near, for all subjects and presbyopic corrections. The ideal correction will have both high near vision and near stereo benefits. We found the largest near stereo benefit for BB (61.92 ± 38.31%; peak at 0.84, followed by FB (22.80 ± 30.12%; peak at 0.02) and FN (17.23 ± 35.21%; peak at 0.04). On average across subjects, near stereo benefit for BB was 0.45 ± 0.38 higher than near stereo benefit for FN, and 0.39 ± 0.42 higher than near stereo benefit for FB, indicating that in most subjects BB provided the best stereo near vision, which on the other hand was compromised in FB and FN.

**Figure 10:**
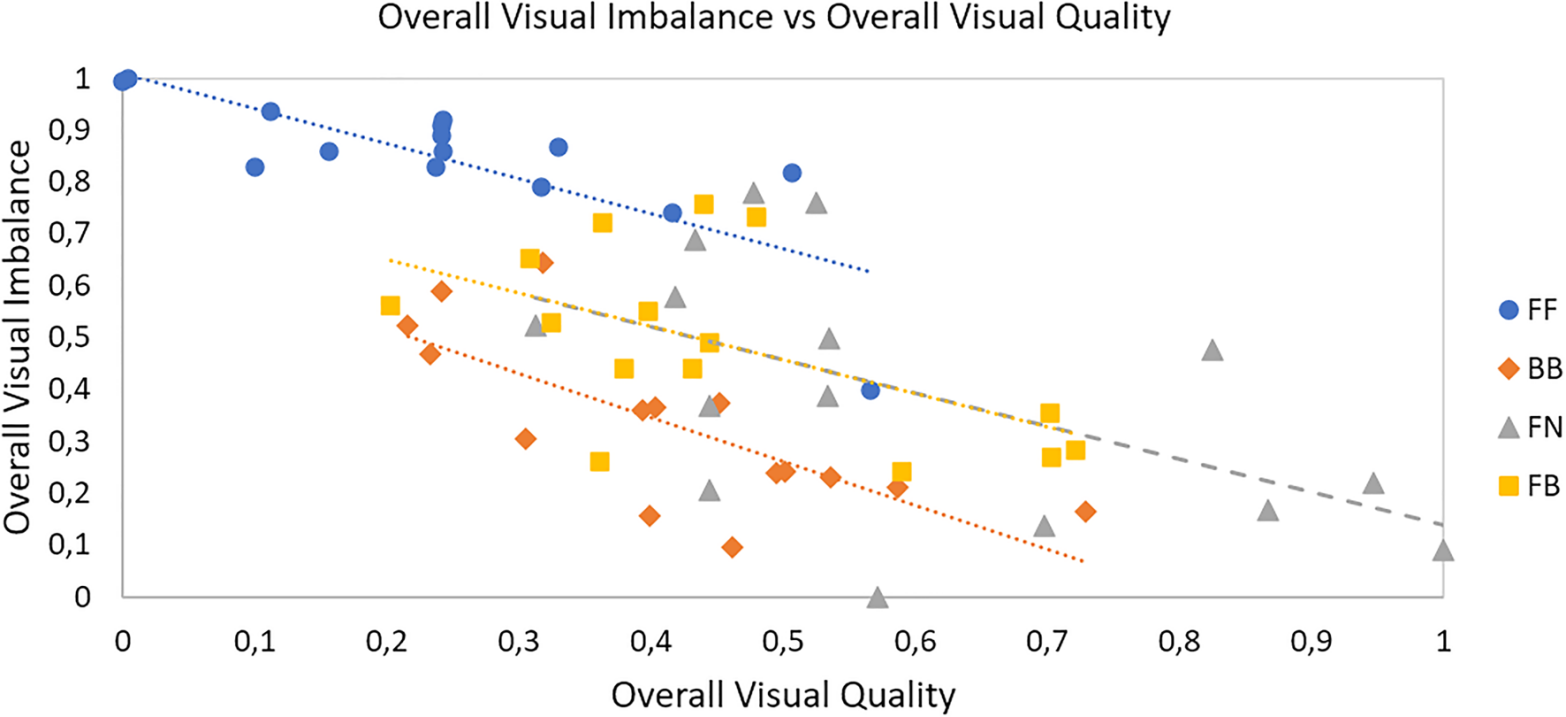
Overall visual imbalance (MAS-2EV polygon asymmetry) vs overall visual quality (MAS-2EV Area), for all tested corrections (far-far; bifocal-bifocal; far-near and far-bifocal).

Figure 10 shows the MAS-2EV overall visual imbalance (polygon asymmetry) as a function of overall visual quality (MAS-2EV Area). For all corrections there was a significant negative correlation between overall visual imbalance and overall visual quality (r = -0.78; p = 0.0005 for FF; r = -0.74; p = 0.002 for BB; r = -0.54; p = 0.04 for FN and r = -0.56; p = 0.03 for FB), indicating that overall quality increases as visual imbalance decreases.

Figures 3, 4, 5, 6, 7, 8, 9 and 10 allow visualizing overall trends with the different corrections and their average performance. However, the parameters at the individual level may have an important predictive power to aid in the selection of a correction or discard corrections not suitable for a patient. Table 2 (for SimVis) and Table 3 (for Contact Lenses) show the ranking of corrections based on the combination (addition) of all parameters: visual degradation at far, visual benefit at near, near stereo benefit, visual imbalance (multiplied by − 1) and overall visual quality (MAS-2EV Area). The corrections marked in bold meet the threshold (0) in the overall performance parameter. The circled number indicates the correction ranked as best for each subject. In 40% subjects with SimVis and 60% subjects with CLs the BB was ranked 1, in 50% (SimVis)/40% (CLs) FN was ranked as 1, and 10% (SimVis)/0% (CLs) FB was ranked as 1.

### Inter- and intrasubject variability

MAS-2EV was highly repetitive across the ten repetitions of each measurement. The intra-subject repeatability of the scoring was consistent in all subjects, with an average standard deviation of 0.97 PS. The standard deviation across repetitions (intra-subject variability) was similar in the different groups (young 1.00 PS; presbyope 0.91 PS), light condition (day 0.99 PS; night 0.93 PS), corrections (FF 0.73 PS; BB 1.05 PS; FN 0.98 PS; FB 1.09 PS) and distances (far 0.78 PS; near 1.14 PS).

However, the inter-subject variability was significantly higher (p < 0.02, paired-sample t-test) for near distance (2.74 PS) than for far distance (1.92), being the difference across distances much higher in young (1.05 PS) than in presbyopic subjects (0.34 PS). Across all subjects, BB at far showed the highest inter-subject variability (1.71 PS).

### Metric repeatability

Given the high repeatability of the responses (Alpha Cronbach’s factor 0.95), we studied the theoretical minimum number of repetitions of a MAS-2EV test that would provide reliable information on the perceived quality of a given correction, and to establish a ranking of corrections. A Repeated Measures ANOVA analysis between factors (for a medium effect size of 0.25) estimated that 3 repetitions provides enough statistical power to show significant differences in perceived quality, influenced by the fixed factors (correction, distance, and light condition). However, a parametric approximation would entail a higher number of subjects per group.

## Discussion

We have presented a new visual metric (MAS-2EV) that captures the multidimensionality of vision, measuring the perceptual quality at different distances, light conditions and stereo. Instead of relying on visual acuity charts or contrast sensitivity targets, judgments of the quality of vision are made on a total of 12 natural images representing habitual visual scenes that the patient can recognize, and that are relevant to his/her daily visual tasks. The test fulfills time requirements in clinical practice both regarding materials (only requires a monitor to display the images at distance and a tablet for images at near, and two LEDs to couple with the night scene), room dimensions (being compatible with the settings of other visual tests) and time of application (evaluation of each correction takes 3 min). The metric has proved repetitive (only 3 repetitions needed to obtain sufficient sensitivity) and provides multiple parameters (including, visual degradation at far, visual benefit at near, near stereo benefit, visual imbalance and overall visual quality) onto which the selection of a correction can be based. The metric is particularly suitable to evaluate and compare multiple presbyopic corrections on the same patients. In the current study, four binocular presbyopic corrections (bilateral monofocal corrections at far, monovision, modified monovision and bilateral bifocal corrections) were evaluated using the MAS-2EV.

The study was conducted on cyclopleged young subjects (i.e. with simulated presbyopia) and a presbyopic group. While presbyopes are the natural users of presbyopic corrections, multifocal corrections have been proposed for myopia control in young patients^31^. In this study, we paralyzed accommodation in the young group, although a similar study could be envisioned for this group also under natural conditions as a way to study vision with these lenses prior to their prescription. Interestingly, on average across groups, we found similar perceptual scores for the same presbyopic corrections and conditions between young and presbyopic subjects (see for example Fig. 5). Only the perceptual scores for the FF correction at near distance were significantly higher in the presbyopic subjects than in the young subjects. This may indicate a higher tolerance to blur at near in presbyopic subjects, likely as a result of neural adaptation^6^. Consequently, the imbalance of far and near vision for FF was significantly higher in young subjects than presbyopes (Fig. 7).

Perceptual scores between day and night conditions (Fig. 6) were small (0.32 PS) but statistically significantly in all groups (p < 0.01). This effect is likely due to the influence of the pupillary dynamics in the study design. On the one hand, measurements in young subjects were performed with a dilated pupil (therefore not reacting to light and potential differences in vergence). On the other hand, the simulations with SimVis were performed for a fixed pupil diameter of 4 mm in all conditions.

MAS-2EV allows a comparison of different presbyopic corrections, including multiple parameters (Figs. 8, 9, 10), and to perform that comparison of the performance of each correction for each subject (Tables 2 and 3). While visual benefit at near was highest for monovision (FN), the inclusion of other parameters such as stereo vision, visual imbalance, and overall quality (MAS-2EV polygon area), increased the value of bifocal corrections (BB). At the individual level, BB resulted the first option in 46.67% of the subjects, FN the first option in 46.67%, and FB in 6.67%.

MAS-2EV allows the evaluation of the visual quality of the real visual world, making it closer to the information collected in the quality of vision questionnaires^13^. However, to date, typical questionnaires are passed after contact lenses are prescribed or after intraocular lens or corneal surgery, and rely, to a large extent, on the patient’s memory, particularly on evaluations of their current quality of vision in comparison with their previous corrections (i.e. monofocal contact lenses or pre-operatively). The value of the MAS-2EV metric presented here is that it is defined to be performed before final prescription, and allows in situ comparison of prospective corrections, while still experiencing a realistic environment which samples different situations of the patient’s visual world (faces, street scenes, theater, driving, navigating a map, reading, halos at night, and stereo tasks).

A goal with the MAS-2EV metric is to aid in the selection of the most suitable correction for a patient. In the current study, the ranking, and hence the optimal correction, is based on a simple addition of parameters, which are blind to the patient lifestyle or preferences. It is conceivable to weight the retrieved parameters with factors accounting for the importance of time spent by the patients on near, far, daytime or nighttime activities, requiring stereovision or benefiting from constant visual quality. The information from these weights could be extracted, from lifestyle questionnaires or devices attached to the patient that measure light exposure and work distance over a period of time^32^.

The MAS-2EV metric has been applied in patients both wearing real contact lenses (monofocal and multifocal, and their combinations) and the Simultaneous Vision Simulator (SimVis Gekko by 2EyesVision) representing monofocal and bifocal contact lenses. The simulation of the exact design of the real contact lens was not attempted. Even with this approximation, the visual experience of the patient with SimVis with all corrections (bilateral far monofocal, bilateral bifocal, monovision and modified monovision) captures to a large extent that provided by similar corrections in the contact lens form, as revealed by the similarity of the MAS-2EV–based parameters obtained with SimVis and contact lenses (Figs. 5, 6). At the individual level (Tables 2, 3) the exact ranking of corrections obtained with SimVis was only strictly the same as with CLs in one subject, although the acceptable corrections (overall performance parameter) were captured similarly by SimVis and by CLs in 70% of the cases. In the young group, where the study was performed with CLs and SimVis, performing the MAS-2EV study with 4 contact-lens based conditions to 2–3 times longer than performing the MAS-2EV with 4 SimVis-simulated conditions (369 vs 164 min per session), as the former requires interchanging of the contact lenses while the latter passes rapidly across the programmed corrections. Similarly, SimVis + MAS-2EV can be used to guide the selection of presbyopic corrections in intraocular lens form, allowing the patient to experience the world with those corrections before implantation. The use of the MAS-2EV metric in combination with visual simulators, such as the SimVis Gekko, therefore serves to reduce chair time in contact lens fitting as well as to reduce uncertainties in intraocular lens surgeries.

In conclusion, MAS-2EV is a suitable metric for its use in the clinic due to its high repeatability, high sensitivity, and short administration time (3 min for subject/condition/correction). Also, the images were designed to be presented in standard displays (or projectors). Further studies are currently underway, including a comparison between MAS-2EV and validated visual quality questionnaires^18,33^, evaluation of the MAS-2EV metric in cataractous patients, evaluation of the sensitivity of the metric in a larger population, evaluation of the metric in patients with other visual limitations and pathologies, study of the effect of pupillary dynamics (in particular, its impact on differences between perceptual scores at near and far) and a direct comparison of perceived visual quality with real contact lenses and their specific programmed design in the visual simulator using MAS-2EV.
